# Mechanisms of Hamstring Strain Injury: Interactions between Fatigue, Muscle Activation and Function

**DOI:** 10.3390/sports8050065

**Published:** 2020-05-18

**Authors:** Shaun Huygaerts, Francesc Cos, Daniel D. Cohen, Julio Calleja-González, Marc Guitart, Anthony J. Blazevich, Pedro E. Alcaraz

**Affiliations:** 1 UCAM Research Center for High Performance Sport, Catholic University San Antonio, 30830 Murcia, Spain; shaun_huygaerts@hotmail.com; 2 Department of Performance, Royal Antwerp Football Club, 2100 Deurne, Belgium; 3 Department of Performance and Health, New York City Football Club, New York, NY 10962, USA; cosfrancesc@gmail.com; 4 National Institute of Physical Education (INEFC), University of Barcelona, 08038 Barcelona, Spain; 5 Masira Institute, University of Santander (UDES), Bucaramanga 680011, Colombia; danielcohen1971@gmail.com; 6 Mindeportes (Colombian Ministry of Sport), Bogota 110311, Colombia; 7 Laboratory of Analysis of Sport Performance, Department of Physical Education and Sport, Faculty of Education, Sport Section, University of the Basque Country, 01007 Vitoria, Spain; julio.calleja.gonzalez@gmail.com; 8 Faculty of Kinesiology, University of Zagreb, 10110 Zagreb, Croatia; 9 Department of Performance, Football Club Barcelona, 08028 Barcelona, Spain; marc.guitart@fcbarcelona.cat; 10 Centre for Exercise and Sports Science Research (CESSR), School of Medical and Health Sciences, Edith Cowan University, Joondalup 6027, Australia; a.blazevich@ecu.edu.au; 11 Faculty of Sport Sciences, Catholic University San Antonio, 30107 Murcia, Spain

**Keywords:** athletic injuries (MeSH), hamstring muscles (MeSH), running (MeSH), biomechanics, muscle functioning, fatigue (MeSH)

## Abstract

Isolated injury to the long head of biceps femoris is the most common type of acute hamstring strain injury (HSI). However, the precise hamstring injury mechanism (i.e., sprint-type) is still not well understood, and research is inconclusive as to which phase in the running cycle HSI risk is the greatest. Since detailed information relating to hamstring muscle function during sprint running cannot be obtained in vivo in humans, the findings of studies investigating HSI mechanisms are based on modeling that requires assumptions to be made based on extrapolations from anatomical and biomechanical investigations. As it is extremely difficult to account for all aspects of muscle-tendon tissues that influence function during high-intensity running actions, much of this complexity is not included in these models. Furthermore, the majority of analyses do not consider the influence of prior activity or muscular fatigue on kinematics, kinetics and muscle activation during sprinting. Yet, it has been shown that fatigue can lead to alterations in neuromuscular coordination patterns that could potentially increase injury risk. The present critical review will evaluate the current evidence on hamstring injury mechanism(s) during high-intensity running and discuss the interactions between fatigue and hamstring muscle activation and function.

## 1. Introduction

Hamstring strain injury (HSI) is the most common non-contact muscle injury in high-speed running sports such as Australian football [1,2,3,4,5], American football [6], rugby [7,8,9] and soccer [10,11,12,13]. This type of injury is characterized by acute pain in the posterior thigh with disruption of the hamstring muscle fibers, where direct external contact with the thigh is excluded as a cause of injury [14,15]. Injury rates are particularly high in soccer, accounting for 37% of all muscle related injuries [16], and a recurrence rate of 12–33% has been reported [13,17,18]. Data showing higher injury rates towards the late stages of each half of a (European) soccer game [16] suggests an association between fatigue and injury risk. Minimizing the risk of first injury is considered a key aspect of overall hamstring injury reduction strategies, as well as secondary prevention [19]. Despite the increasing research focus in this area, the potential injury mechanisms are not well defined [20], and injury incidence seems to have either remained about the same [15] or even increased (e.g., soccer; [21]) in recent years. This injury burden is a concern for clubs in terms of team performance from a key player availability perspective in senior professionals [22] and in the long-term development of younger players [23]. It is also associated with a significant financial cost (e.g., the average cost for a first team player is approximately €500,000 per month in European soccer leagues) [24]. For these reasons, there remains considerable interest among researchers and practitioners in investigating hamstring injury mechanism(s) in order to develop evidence-based risk reduction prevention strategies. 

Neuromuscular fatigue is one potential major HSI risk factor [15,25,26]. Fatigue can be defined as the inability to maintain a given exercise intensity or power output, resulting from either acute or residual (i.e., inadequate recovery from repeated exposures to load) physical exercise burden [27]. The acute fatigue that develops during and immediately after the conclusion of bouts of physical activity is attributed to a combination of central and peripheral fatigue mechanisms [27]. Central fatigue affects the voluntary activation of muscle and principally occurs during submaximal, low-intensity muscle contractions [28]. It can be caused by a decrease in the excitation supplied by the motor cortex and/or a decrease in motoneuronal pathway activity [28]. Peripheral fatigue corresponds to an alteration in muscle contraction capacity and can be induced by disturbances in the propagation of the muscle action potential, excitation-contraction coupling and contractile (force production) mechanisms [28]. In sports such as soccer, mechanical demands, particularly those related to decelerations and eccentric actions, can induce muscle damage. This promotes increases in inflammatory proteins and immune cells and subsequent alterations in redox status during the recovery period after a game [29]. The exercise-induced muscle damage and its sequelae can reduce physical performance for up to several days [30] and are suggested to be a principle cause of the residual fatigue observed in soccer players following competition [27]. While evidence for an association between acute fatigue and injury is principally epidemiological [16], there are a number of viscoelastic, neuromuscular and biomechanical alterations demonstrated during simulated/real competitions that could theoretically increase susceptibility to injury. The accumulation of fatigue across training and competition periods is also associated with elevated injury risk, especially when abrupt increases in total training loads or intensities occur [31] or during periods of match congestion [32]. Nevertheless, acute, residual and chronic fatigue might influence risk in very different ways, and consequently, the strategies used to reduce the risk associated with each may differ. Since player monitoring practices have the potential to reduce residual fatigue-related injury risk [33], monitoring of player workload and response to workload—in the form of subjective and objective assessment of fatigue—is now commonly performed and is considered a cornerstone of player welfare systems [33]. However, the present article will primarily consider the potential effect of acute fatigue on associated risk factors for HSI. Therefore, the objective of this critical review is to evaluate the current evidence and provide an overview of: (1) mechanisms of hamstring injury and (2) interactions between fatigue, hamstring muscle activation and function and potential interactions with risk.

## 2. Mechanisms of Hamstring Strain Injury

Animal models have been used to try and determine the general mechanisms of muscle strain injury, suggesting that excessive muscle strain in eccentric contractions or stretching is the main mechanism of muscle strain injury [34]. Two specific hamstring injury types, defined by the injury mechanism, have been described: (1) stretch-type and (2) sprint-type [35]. The stretch-type hamstring injury occurs in movements involving a combination of extreme hip flexion and knee extension (e.g., kicking and dance maneuvers), while the sprint-type injury occurs during maximal or near-maximal running actions [18]. Both injury types are strain injuries; however, the stretch-type seems to occur at long muscle lengths, while the sprint-type may occur well within the normal working range of the muscle [18]. Using magnetic resonance imaging (MRI), the stretch-type hamstring injury has been shown to primarily affect the semimembranosus, and particularly the proximal free tendon rather than the intramuscular tendon [36,37]. In contrast, the sprint-type hamstring injury primarily involves the long head of biceps femoris (BFlh) [23,38]. Injuries to BFlh show a greater involvement of the proximal region compared to the distal region [23,39], with the musculotendinous junction (aponeurosis and adjacent muscle fibers) reported as the most common injury location [23,38,40].

Since the majority of HSIs occur during maximal or near-maximal running efforts [23,37,39], the present critical review will focus on the sprint-type injury. The phase of the running cycle during which HSIs most commonly occur [41] remains a controversial topic in sprint-type HSI research. In order to increase the efficacy of hamstring injury risk reduction strategies, a complete understanding of the biomechanical function of the hamstring muscles during sprinting is required [41]. In the following sections, we outline the current evidence relating to HSI mechanisms and examine the possible influence of fatigue and the interactions between fatigue, hamstring muscle activation and function. 

### 2.1. Hip, Knee and Hamstring Mechanics during High-Speed Running

Before exploring the mechanism(s) of sprint-type injury, the running cycle should be described. A complete running cycle includes two main phases: the stance phase (foot in contact with the ground) and the swing phase (foot not in contact with the ground). These two main phases can be further divided into sub-phases: early stance (braking), late stance (propulsion), early and middle swing (recovery) and late swing (pre-activation) [42]. In a recent review, Kenneally-Dabrowski et al. [43] comprehensively described hip, knee and hamstring mechanics during high-speed running, showing that during the stance phase net joint torque mainly results from muscle torques (generated from muscle contractions) and external forces (resulting from ground reaction forces) [43]. On the other hand, during the swing phase net joint torque mainly results from muscle torques and motion-dependent torque (resulting from the mechanical interaction of segments) [43]. The hip displays extensor dominance (reaching peak extension torque at approximately 4.1 N·m·kg^−1^) during the early stance phase and shifts to a flexion moment towards the latter half of stance [43]. Regarding knee moments, Schache and colleagues [44] reported an extension moment for the first half of the stance phase (peaking at 3.6 N·m·kg^−1^) before a flexion moment is produced towards the late stance phase [43,44,45]. However, other studies have reported much more variable knee moments, sometimes switching several times from extension to flexion dominance throughout the stance phase, findings which may be attributed to different filtering techniques utilized and data processing [43]. The hip displays a large flexion moment during the first half of the swing phase (peaking at 4.3 N·m·kg^−1^), while the knee produces a small extension moment (1.0 N·m·kg^−1^) [44,45]. During the second half of the swing phase, the hip displays a large extension moment (4.2 N·m·kg^−1^), while the knee produces a smaller flexion moment (1.8 N·m·kg^−1^) [44,45]. Furthermore, the hamstring muscles undergo a stretch-shortening cycle throughout high-speed running actions [46,47]. Traditionally, it has been suggested that BFlh shortens during the first part of the swing phase as the knee flexes and the hip moves from extension to flexion [43]. Subsequently, a rapid lengthening of BFlh takes place as the hip continues to flex, while the knee extends throughout the second half of the swing phase [43]. Next, BFlh starts to shorten as the hip extends and the knee flexes in preparation for foot strike [43]. Throughout the stance phase, the hip continues to extend and the knee flex for the first half, before starting to extend [43]. While most studies suggest that BFlh shortens throughout the stance phase, two studies have reported a lengthening of the hamstrings during the late stance phase [48,49]. It is therefore suggested that length change could be dependent upon the degree of hip and knee extension exhibited by an individual athlete [43]. In reality, this movement pattern would be influenced by the athlete’s anatomical variability [50] and specific sprint mechanics [51].

### 2.2. The Late Swing Phase 

The majority of researchers investigating the role of the hamstrings during sprint-running argue that the late swing phase is likely to be the point in the running cycle at which the hamstrings are most susceptible to injury [45,52,53,54,55,56]. Indeed, during sprinting, maximum electromyogram (EMG) activity has been consistently shown to occur during the terminal swing phase [52,55,57]. Furthermore, based on their modelling of muscle force and length characteristics during sprint running, Chumanov et al. [52] concluded that the hamstring muscle-tendon unit exclusively undergoes a lengthening activation during the late swing phase. Furthermore, Schache et al. [45] identified peak muscle-tendon strain, peak muscle-tendon force and the occurrence of negative work in BFlh, semitendinosus (ST) and semimembranosus (SM) during the late swing phase during maximal sprint running. More specifically, BFlh displayed the largest peak muscle-tendon strain (12.0% increase in length from upright stance position), ST displayed the greatest muscle-tendon lengthening velocity, and SM produced the highest muscle-tendon force, absorbed and generated the most muscle-tendon power and performed the largest amount of positive and negative work. Additionally, Higashihara et al. [55] found that peak muscle-tendinous stretch was synchronous with the peak EMG activation in BFlh during the late swing phase in overground sprinting [55]. As running speed increased from 80% to 100%, biceps femoris (BF) activity during the terminal swing phase increased an average of 67%, while ST and SM only showed a 37% increase [39]. Speculatively, these disproportional increases in the demand on BF at maximal running speeds may also contribute to its greater tendency to be injured during high-speed running than the other hamstring muscles. However, a study using finite-element computational modeling based on muscle-tendon dimensions of athletes participating in high-speed sports reported that whole-fiber length change of BFlh relative to the muscle-tendon unit length change remained relatively constant as running speed increased [53]. Nonetheless, the computational models also predicted that peak local fiber strain relative to the strain of the muscle-tendon unit increased with speed, with the highest peak local fiber strain relative to the whole muscle fiber strain occurring at the fastest speed (100% maximum) during the late swing phase [53]. These findings, and the observations of two independent hamstring injury case studies [54,56], support the notion that injury susceptibility is greater during the late swing phase. However, it should be noted that the majority of analyses based on mechanical models did not consider the potential additional influence of prior activity and fatigue on kinematics, kinetics and muscle activation during sprinting.

Some issues need to be highlighted before interpreting these studies. First, detailed information relating to hamstring muscle function during sprint running cannot be obtained in vivo in humans. The findings of studies investigating hamstring strain injury mechanisms are therefore principally based on modeling and assumptions extrapolated from anatomical and biomechanical investigations [58]. With these simulation models, it remains extremely difficult to account for all aspects of muscle tissue that influence function during high-speed running actions, and as such, much of this complexity is omitted. For example, Van Hooren and Bosch [59] speculated that the hamstring muscles do not actively lengthen during sprinting and that it is more likely that the hamstrings function predominantly isometrically during the swing phase of running [59]. However, the authors also hypothesized that fascicle lengthening may feasibly underpin muscle injury, but that this eccentric action may occur as a result of the inability of fascicles to remain isometric [59]. Although these arguments were mainly based on evidence from animal studies, their work has challenged the conclusions of current modeling studies investigating the mechanism of hamstring injury during high-speed running. According to Van Hooren and Bosch [60], potential errors in modeling studies would include an underestimation of stretch in the tendinous tissues, and hence overestimation of fascicle stretch as a result of potential differences in real and modeled tendon stiffness, as well as not accounting for muscle slack (i.e., compliance)—causing a delay between muscular contraction and recoil of the series elastic elements [61]. Secondly, as mentioned previously, given the epidemiological evidence, the presence of fatigue is likely a key factor in relation to HSI susceptibility. Since fatigued muscles seem to be able to absorb less energy before reaching the stretch limit, fatigue may directly increase risk of injury [62]. Therefore, it is logical to consider interactions between fatigue and muscle activation and function as a potential mediator of risk, as will be further outlined below. Yet, the majority of studies investigating hamstring muscle mechanics in relation to HSI susceptibility have examined sprint running in non-fatigued conditions (Table 1) [20,41,45,47,49,52,53,54,55,56,57,63,64,65,66].

### 2.3. The Early Stance Phase

Although HSI is commonly thought to occur in the terminal swing phase, and taking into account that this assumption has been established without full consideration of the problems raised above, the possibility cannot be excluded that HSI can also occur in the early stance phase, during which some studies report a second peak in hamstring muscle activity [49,52]. In fact, decades ago researchers speculated that the early stance phase would be the highest risk phase of sprint running [64,65]. This hypothesis was based on the finding that maximum hip extension and knee flexion torques occurred during ground contact [64,65]. Furthermore, although peak BFlh muscle-tendon force has been reported to occur during the late swing phase, a second (smaller) peak has also been observed during the early stance phase [45,46]. Some researchers have suggested that BFlh muscle-tendon force in the early stance phase may be underestimated due to over-filtering of force and kinematic signals, which could potentially result in erroneously low hip and knee torques [20,41]. During the early stance phase, it is also suggested that the ground reaction force causes a large extension torque at the knee and a flexion torque at the hip [43]. Consequently, in order to counter the large passive forces, the hamstring muscles would need to produce large flexion torques at the knee and extension torques at the hip, placing them under an extremely high load [20,41]. In addition, using EMG and MRI measurements, selective recruitment of ST and gracilis has been observed with lengthening during knee extension [67]. In addition, BFlh and SM may be selectively recruited during tasks dominated by hip extension such as standing, forward bending or extending backward from the hip [68]. Based on these data, it could be speculated that acute cases of HSI involving BFlh could also occur during the stance phase of sprinting. 

Furthermore, the results of a study which calculated a surrogate measure of hamstring tensile forces during overground sprinting using EMG, ground reaction force and 3D motion analysis data, did support the early stance phase as a point of increased susceptibility to HSI [66]. The product of normalized muscle-tendon length and the normalized EMG (nEMG) value was calculated for each muscle and defined as the tensile force index [66]. The tensile force indexes of BFlh and ST increased abruptly in concert with the largest magnitudes noted within the stance phase [66]. Moreover, during the period from the foot strike to the peak ground reaction force in the early stance phase (lasting approximately 0.01 s), BFlh nEMG reached its peak when the knee joint was extended maximally [66]. These findings, combined with evidence that greater muscle activation and larger muscle forces may be associated with higher risk of muscle strain [63,69], suggest that BFlh would be susceptible to strain injury during the early stance phase of the sprinting stride. However, as the relevance of tensile force index to injury risk has not been established, the conclusions of this study should be considered with caution [66], highlighting again the issues of using simulation models to analyze and interpret hamstring tissue mechanics. 

### 2.4. The Swing-Stance Transition Period

**Figure 1:**
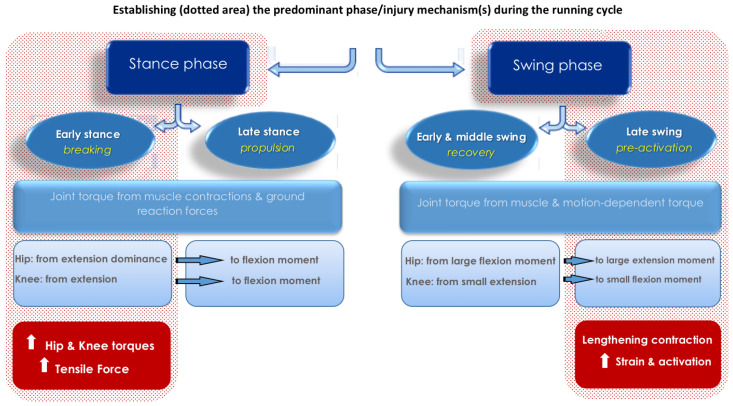
Mechanical and muscular activation characteristics of the main phases in which HSI occurs.

One recent study suggested that the risk of sustaining HSI is high in both the late swing and the early stance phases but with different loading mechanisms underpinning them [41]. Liu et al. [41] concluded that large passive torques at the knee and hip joints during maximal sprinting in elite athletes acted to lengthen the hamstring muscles in both phases. The active muscle torques generated mainly by the hamstrings counteracted the passive effects generated by the forward swing of the leg (late swing phase) and the external ground reaction force (early stance phase) [41]. As a result, the researchers suggested that these two phases may be considered to be a single phase (the swing–stance transition period) (Figure 1) because lower extremity joint motions are continuous and the hamstring muscles function to extend the hip and flex the knee throughout the entire phase [41]. 

At present, the evidence is inconclusive as to which phase in the running cycle has the greatest HSI risk, although the possibility that both late swing and early stance phases may both put excessive loads on the hamstring muscles with consequent increases in injury susceptibility cannot be discounted. Importantly, better models are required in order to elucidate hamstring muscle-tendon behavior and functioning in both phases as well as a need for the evaluation of changes in the kinetic, kinematics, EMG activity and modeling under fatiguing conditions.

### 2.5. Interactions between Fatigue, Hamstring Muscle Activation and Function

Differences in the changes in muscle activation with increasing running speed have been observed between hamstring muscles within the running cycle [57]. During sprint running (speeds above 95%max), ST EMG activity was significantly greater than that of BF during the middle swing phase, but not during the early stance or late swing phases when the activity of both muscles increased to a similar extent [57]. However, an earlier peak activation time in ST than BF was observed during the late swing phase of sprint running, while an earlier peak activation time in BF compared to ST was seen during the stance phase [57]. Thus, it appears that BF is activated earlier than ST to prepare for high impact moments at foot strike when running close to maximum sprinting speed, which could be explained by the important role of BF in the generation of the forward propulsive force [57]. These temporal differences in activation patterns between the hamstring muscles may suggest complex neuromuscular coordination patterns during the running cycle that vary as running speeds increase [57]. Given that the BF and ST muscle bellies share a common proximal tendon origin, differences in activation patterns and the timing of peak activation between these muscles may exert significant influence on HSI risk [57]. In accordance with this, Avrillon et al. [70] observed individual-specific differences in hamstring muscle coordination strategies and hypothesized that these individual muscle coordination strategies might have functional consequences. For example, if one muscle is activated to a greater extent than required by the task, which is inevitably the case when activation between synergists is imbalanced, its metabolic demand would be higher and fatigue would develop sooner, which the authors suggested could increase injury risk [70]. Conversely, fatigue influences muscle activation patterns during maximum sprint running [71] so that neuromuscular coordination is altered under fatiguing conditions. It is suggested that this could place an excessive load on neighboring tissues, which could induce excessive tensile shear stress and potentially increase injury risk [72]. 

Differences in muscle activation patterns have also been associated with lower limb kinematic changes [73]. In sprints performed after hamstring-specific fatiguing exercise, an earlier reduction in rectus femoris activity and earlier onset of ST and BF activities was observed in the sprint cycle [71]. This was suggested to contribute to observed kinematic changes, including decreased hip flexion and increased knee extension at the point of maximum knee extension in the swing phase, decreased leg angular velocity immediately before foot strike and decreased angular displacement of the trunk, thigh, and leg segments during the late swing phase of the sprint cycle [71]. The observed decreases in angular displacement of the trunk, thigh and leg segments in the late swing phase were attributed to the decreased hip flexion and increased knee extension at the point of maximum knee extension in the swing phase [71]. Small et al. [73] studied the changes in sprinting mechanics after simulating the physiological and mechanical demands of soccer match play and observed reduced maximum hip flexion and knee extension angles during the late swing phase, an increased anterior pelvic tilt and an increased lower limb segmental velocity [73]. An increased anterior pelvic tilt has the potential to increase BFlh length while running, since BFlh attaches directly to the ischial tuberosity of the pelvis [74]. Therefore, the muscles controlling hip motion and pelvic position could influence the relative BFlh length during running [74]. The increase in leg angular velocity, which contrasts with the findings of Pinniger et al. [71], was suggested to be linked to an impaired ability of the hamstrings to decelerate the limb effectively [73]. Indeed, soccer-specific fatigue has been associated with significant increases in peak eccentric isokinetic knee flexion torques [75,76], with decrements greatest at longer hamstring muscle lengths [76]. These findings suggest that the force absorption capacity might be particularly reduced at the longest hamstring muscle lengths, which could increase vulnerability to hamstring muscle strain since force production in sprint running is highly dependent on the utilization of recoil energy from elastic tissues [77]. Both Pinniger et al. [71] and Small et al. [73] interpreted the decreased thigh flexion angles as a change that might form part of a protective mechanism to reduce stress on the hamstring muscles at critical phases of the stride cycle. They also reported contradictory findings regarding knee joint angles and leg angular velocity, which could possibly be explained by the different fatigue protocols employed by these research groups. In support of this theory, Hader et al. [78] observed a greater reduction in hamstring muscle activity during intermittent high-intensity efforts integrating 90° changes of direction compared to straight-line high-intensity running, despite the lower running speeds observed in the 90° changes of direction [78]. It seems that the differing hamstring muscle demands and fatigue profiles imposed by straight line running and changes of direction may, therefore, also have different outcomes on high-speed/sprint running biomechanics, which is highly relevant to soccer and other multi-directional repeated sprint sports. 

The hamstrings are also reported to play a crucial role in dynamic knee stability and control, helping to maintain joint integrity [79,80]. Along with several other complex ligamentous and musculotendinous structures (e.g., iliotibial band, biceps femoris short head, lateral collateral ligament, popliteus muscle/complex, lateral gastrocnemius tendon, joint capsule/mid-third lateral capsular ligament, coronary ligament of the lateral meniscus, oblique popliteal ligament and the fabellofibular ligament), the distal BFlh tendon forms part of the posterolateral complex of the knee since it attaches to the head of the fibula and the lateral condyle of the tibia [81]. The posterolateral complex has been described as a critical element for lower extremity stability [81]. In accordance with this, a recent study by Cleather [82] provided support to the theory that the hamstring muscles play an important role in dynamic knee stability and control, creating rotational stability of the tibia in the transverse plane [82]. Therefore, speculatively, increased knee stabilizing demands (e.g., change of direction actions) and the presence of fatigue could place greater loads on the hamstring muscles, thereby increasing their vulnerability to strain injury.

**Figure 2:**
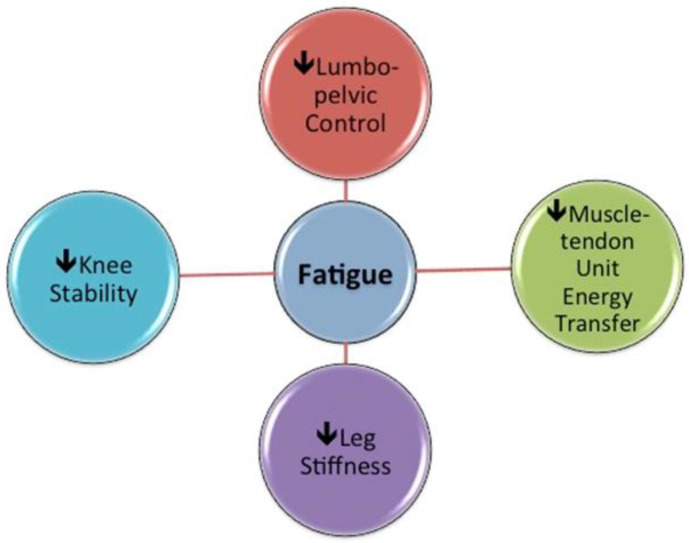
Interactions between fatigue and hamstring muscle activation and function.

It has been suggested that decreased lower limb muscle stiffness observed during fatiguing stretch-shortening cycle exercises leads to a decrease in the amount of stored and reused elastic energy [83]. Lehnert et al. [84] found significant reductions in leg stiffness (decreased hip, knee and ankle flexion measured in repeated two-legged hopping) and reactive strength (the flight-to-contact time ratio measured in drop jump) after a 90-min soccer-specific aerobic field test protocol (SAFT^90^—a series of soccer-specific fatiguing exercises incorporating utility movements and frequent accelerations and decelerations, as is inherent to match play [73]) in young footballers [84]. In addition, based on in-match accelerometer data and weekly monitoring of jump performance in Australian football players, Cormack et al. [85] suggested that neuromuscular fatigue was associated with a reduction in vertical acceleration in subsequent competition and speculated that this outcome resulted from the inability of the neuromuscular system to maintain vertical stiffness [85]. These changes in turn promote the adoption of a “Groucho” running pattern, a form of locomotion in which the knees remain flexed during the complete stride. This reduces vertical ground reaction forces (including impact forces) but increases the energy cost of locomotion by minimizing the elastic bounce of the body and thus the energy savings that would normally come from bounce-like gait and is associated with decreased running speeds, reduced acceleration/deceleration abilities and greater O_2_ consumption [85,86]. The alterations in running kinematics, reduced movement efficiency and greater moments of force are associated with an increased load on the contractile muscle units, theoretically increasing strain injury risk (Figure 2).

Furthermore, evaluation of EMG relative to distance covered indicates that BF and rectus femoris fatigue occurs earlier than other lower extremity muscles such as vastus lateralis, gastrocnemius and tibialis anterior during high-speed, but not low-speed running [87]. This may be linked to the higher activation as running speed increases [39], underpinned by the dominant role of BF in hip extension [66] and the importance of hip extensors for horizontal force production during sprinting [88]. In line with this, a fatiguing repeated-sprint protocol on a motorized instrumented treadmill resulted in decreased sprint acceleration (maximal power output) and horizontal force production [89]. In addition to decreased maximal power output and horizontal force production, the researchers found (i) a higher horizontal force production in the fatigued state that was mainly associated with a higher concentric peak torque of the hip extensors, (ii) a smaller decrease in horizontal force production after fatigue that was mainly associated with a lesser reduction in gluteus maximus activity at the end of the swing phase and (iii) that while hamstring muscle torque during knee flexion was associated with horizontal force production in the non-fatigued state, this association was not observed in the fatigued state [89]. The authors suggested that the decrease in maximal power output under fatigue could be linked specifically to the decrease in horizontal force rather than the total force output and that this partly resulted from reduced hamstring muscle function [89], promoting a compensatory increase in the contribution of other hip extensors such as gluteus maximus [89]. 

The altered hamstring muscle function could also be linked to the previously mentioned “Groucho position” consequent to fatigue, resulting in a less efficient muscle-tendon unit energy transfer in BFlh and increasing the demand on adjacent structures and/or synergist muscles such as gluteus maximus. There is also a growing body of evidence indicating that lumbo-pelvic muscle function may play an important role in HSI risk reduction [90,91]. Indeed, the presence of fatigue has been shown to promote anterior pelvic tilt in soccer players, potentially predisposing them to increased injury risk due to increasing relative BFlh length [73]. Furthermore, it is well established that eccentric knee flexor strength training interventions can decrease HSI risk [92], while Delextrat et al. [93] also showed that training with a strength-endurance emphasis significantly reduced the decline in hamstring eccentric peak torque associated with simulated match play [93]. In addition, there is retrospective evidence that prior HSI is associated with strength-endurance deficits [94] and prospective evidence that a weaker test score on a hamstring specific strength-endurance test (single leg hamstring bridge) is related to greater HSI risk [95]. Therefore, the effects of strength-endurance training interventions of both the hamstrings and their synergists on HSI risk warrant further investigation. 

## 3. Conclusions

In conclusion, the hamstring muscles generate large opposing forces during high-speed running [96] while also playing a role in the production of dynamic stability at the knee [82]. Based on the present research, the possibility exists that changes in muscle coordination strategies may cause one or more hamstring muscles to be disproportionately activated [70], possibly increasing metabolic demand and thus prematurely fatiguing the overactive muscles [70]. With fatigue it appears that lower limb stiffness decreases [85], which could cause the adoption of a “Groucho” running pattern, associated with reduced movement efficiency and greater joint moments of force. This phenomenon, in combination with increased anterior pelvic tilt (due to lumbo-pelvic instability) during running, could potentially place BFlh at a relatively longer length where it is more vulnerable to strain injury [51,90,91]. Hence, the late swing and early stance phases appear to be critical points at which HSI is more likely to occur. More accurately defining the influence of fatigue on tissue behaviors in these two phases may be key to gaining a better understanding of hamstring injury mechanisms. 

## 4. Future Directions

Building on the current understanding of hamstring mechanics and acknowledging that the occurrence of injury is likely to be multi-factorial, several theories can be proposed that warrant testing. A significant limiting factor in current research is that simulation models used to estimate tissue behavior of the hamstring muscle-tendon are incomplete, and possibly oversimplified. This results from our limited understanding of the mechanical capacities of numerous tissues that influence system function, difficulties in measuring muscle activity states and timing and inevitable issues around the indeterminacy of the system. Importantly, the current models do not account for anatomical variability between individuals and the behavior and influence of the surrounding tissues. In addition, the majority of modeling studies do not test for the influence fatigue, which is likely to be a major HSI risk factor. In relation to this, some recent evidence highlights the need for further investigation of the role of strength endurance in HSI risk. Future research would require the development of better simulation models, especially using data obtained under fatiguing conditions. In addition, prospective studies are required that take into consideration the neuromuscular function of a number of synergists and characterize not only peak muscular strength, but also fatigue resistance in these muscles.

## Figures and Tables

**Table 1:** Summary of studies using biomechanical models to estimate the phase of the running cycle at which the hamstring muscles are most susceptible to strain injury.

Reference (Year)	Participants	Late Swing Phase	Early Stance Phase	Late Stance Phase
Schache et al. (2012) [45]	Sprinters (5 males, 2 females)	X		
Chumanov et al. (2011) [52]	Recreational athletes (9 males, 3 females)	X		
Fiorentino et al. (2014) [53]	Track and field athletes (7 males, 7 females)	X		
Higashihara et al. (2014) [55]	Track and field athletes (13 males)	X		
Higashihara et al. (2010) [57]	Track and field athletes (8 males)	X		
Thelen et al. (2005) [63]	Recreational athletes (9 males, 6 females)	X		
Thelen et al. (2005) [47]	Recreational athlete (1 male)	X		
Yu et al. (2008) [49]	Sprinters or middle-distance runners (20 males)	X		X
Mann and Sprague (1980) [65]	Sprinters (15 males)		X	
Mann (1981) [64]	Sprinters (15 males)		X	
Ono et al. (2015) [66]	Track and field, rugby and soccer players (12 males)		X	
Sun et al. (2015) [20]	Sprinters (8 males)	X	X	
Liu et al. (2017) [41]	Sprinters (8 males)	X	X	
* Schache et al. (2009) [56]	Australian Rules Football player (1 male)	X		
* Heiderscheit et al. (2005) [54]	Skier (1 male)	X		

Note: The modeling studies included in this table obtained measurements in non-fatigued participants. * Case studies of hamstring strain injury (HSI) occurrence during assessment.
